# Characterizing children’s eating patterns: does the choice of eating occasion definition matter?

**DOI:** 10.1186/s12966-021-01231-7

**Published:** 2021-12-19

**Authors:** Rebecca M. Leech, Alison C. Spence, Kathleen E. Lacy, Miaobing Zheng, Anna Timperio, Sarah A. McNaughton

**Affiliations:** Institute for Physical Activity and Nutrition, Deakin University, Geelong, Victoria Australia

**Keywords:** BMI, Children, Eating occasions, Eating patterns, Meal frequency, Snack frequency

## Abstract

**Background:**

Recommendations to define eating occasions (EO) currently exist for research in adults, but not for children or adolescents. We examined how varying EO definitions affect the characterization of eating patterns in children and adolescents.

**Methods:**

Cross-sectional dietary data collected using a 24-h recall data during the 2011–12 Australian National Nutrition and Physical Activity Survey (1364 boys and 1337 girls aged 2–18 years) were analyzed. Eight definitions were applied: participant-identified, time-of-day, and 6 neutral definitions (EO separated by 15- or 60-min and/or an additional energy criterion of 21 or 210 kJ). Frequency of and total energy intake from meals, snacks, and all EO were estimated. *F* tests stratified by gender and age-group, were used to assess differences between definitions. Agreement between definitions of meal and snack frequencies was assessed using intraclass correlation coefficients (ICC). Linear regression was used to estimate the proportion of variance in total energy intake (kJ) and BMI z-score predicted by each definition.

**Results:**

Mean frequencies of meals and snacks differed between the participant-identified and time-of-day definitions, in boys and girls and for all age groups (*P* < 0.01). Across the six neutral definitions, there were differences between mean frequencies of EO with the largest mean difference observed for children aged 2–3 y (boys: 2.3, girls: 2.5; *P* < 0.003). Between the participant-identified and time-of-day definitions, there was good agreement for frequencies of snacks (ICC for both genders: 0.93) but not meals (boys: 0.36; girls: 0.38). The 15-min time interval plus 210 kJ definition of an EO consistently predicted the most variance in total energy intake (R^2^ range = 8.1–34.8). Definitions that delineated meals and snacks better predicted variance in BMI z-score, when compared to the neutral definitions.

**Conclusions:**

How eating patterns are characterized vary depending on the EO definitions employed, particularly in young children. Variance in total energy intake was best predicted by a variation of the neutral definition whereas definitions that delineated meals and snacks performed better in relation to predicting BMI variance. Further international research that compares EO definitions in children will help inform a standard approach.

**Supplementary Information:**

The online version contains supplementary material available at 10.1186/s12966-021-01231-7.

## Introduction

There is increasing interest in understanding the dietary and health implications of different eating patterns [1–5]. Eating patterns may describe the frequency, temporal distribution and nutritional content of eating occasions (EO) [1]. EO include meals and snacks and represent opportunities for translating the dietary recommendations and aligning dietary intakes with the current dietary guidelines. As such, EO are the fundamental intermediary steps for achieving a diet that promotes optimal health and wellbeing [6].

Dietary habits tend to track over the life course and eating patterns during childhood are likely to be important for short and long-term health [7–9]. For example, frequency of shared/family meals are linked to better diet quality in boys and girls [10]. On the other hand, snack frequency is associated with higher intakes of discretionary (i.e., energy-dense and nutrient poor) food and beverages [11–14] which may promote dental caries and obesity [9]. Evidence from cross-sectional studies suggest that skipping breakfast and EO frequency are positively [15, 16] and negatively [5] associated with children’s adiposity markers (e.g., BMI), respectively. However, not all cross-sectional studies have found an association and longitudinal evidence to support a relationship between eating patterns and adiposity is lacking [3, 6]. Further, a key criticism of eating patterns research, exemplified in the breakfast skipping and EO frequency literature, is the variation across studies in how EO are defined. These inconsistencies have hampered study comparisons of children’s eating patterns [3, 6] and highlight the need for a standardised definition of an EO [1].

Whilst there is now an evidence-based recommendation for defining EO in studies of adults [2, 3], the choice of EO definition in children or adolescent study populations has received little attention, despite a variety of definitions being used to date [14, 17–20]. Some studies have classified EO into meals and snacks based on participant-identification of the EO type or the time-of-day [14, 17]. Other studies have employed a neutral approach whereby meals and snack labels were not assigned to specific EO. Instead, EO are delineated using criteria based on time and/or energy content [17–20]. How EO are defined has been shown to affect how eating patterns are quantitatively characterised (e.g., total frequency of meals, snacks and EO) [21]. However the recommended definition for use in adults [2, 21] that applies a 15-min interval and minimum energy content of 210 kJ to delineate individual EO may not be suitable for younger children with differing appetite and satiety determinants and need to eat smaller amounts more frequently [22]. Finally, age-appropriate definitions should also consider how meals and snacks are classified (e.g., time-of-day vs participant identified) as adherence to conventional mealtimes [22] and the distribution of dietary intake across the main meals of the day may change with increasing age and independence [7, 23, 24].

Although researchers can choose from multiple definitions to characterize children’s eating patterns, there is a dearth of evidence to guide which definition to use that are age appropriate. Standardized approaches to defining EO and classifying EO types will facilitate understanding of associations between eating patterns, dietary intake, and health outcomes, and the implications of varying definitions. Therefore, the aim of this study was to understand how different EO definitions affect the characterization of eating patterns among Australian children and adolescents. We also examined variance in total energy intake, weight of food intake and BMI-for-age z-scores predicted by the frequencies of EO, meals and snacks, for each definition.

## Methods

### Sample and study design

The study was a cross-sectional secondary analysis of data from the 2011–2012 National Nutrition and Physical Activity Survey (NNPAS). The nation-wide survey, conducted by the Australian Bureau of Statistics (ABS) from May 2011 to June 2012, employed a multistage, stratified area, probability sampling design to recruit participants from private households. The methodology of the survey has been described in detail previously [25]. Private households in urban and rural areas of Australia were eligible for inclusion in the survey. Non-private households (e.g., hostels, boarding houses, nursing homes), households in very remote areas of Australia and those which contained members of non-Australian defence forces (stationed in Australia), or diplomatic personnel of overseas governments were excluded from the survey [25]. For each responding household, one adult (a usual household resident aged 18 years and older), and where applicable, one child aged 2–17 years, were randomly sampled. The final sample included 9519 responding households (77% household response rate) from which 9435 participants aged 18 years and over and 2718 participants 2–17 years were selected, respectively. For this study, data on 2812 children and adolescents aged 2 to 18 years were used (Additional File 1). During a household interview, information was collected on participants’ dietary intakes, and sociodemographic and health characteristics. Ethics approval was provided by The Census and Statistics Act 1905 and allows the ABS to conduct all household interview and measurement components of Australian surveys [25].

### Dietary assessment

Information on child and adolescent diets were collected during an in-person 24-h recall interview which employed the validated US Department of Agriculture automated multiple-pass method [26, 27]. The dietary recall interviews were conducted by trained ABS staff, occurred on days that suited participants and covered all days of the week and all months of the year. The person reporting the dietary recall varied by age group. For children aged 2–11 years, an adult proxy was used for the interview; however, children aged 6–8 years were encouraged to assist in answering the questions. Interviews for children aged 9–11 years were proxy-assisted whereas children aged 12–14 were interviewed directly with a proxy in the room. For adolescents aged 15–17 years, permission was sought from their parent/guardian to conduct the dietary recall without the assistance of a proxy. A second dietary recall was conducted during a telephone interview approximately 9 days later but only included a subset (60%) of the sample [25]. A food model booklet containing a ruler guide and photographs/drawings of foods and food containers was used by the interviewers to help participants and/or proxies with portion size estimation. All food and beverages reported at each EO were uniquely coded and the Australian Supplement and Nutrient Database (AUSNUT) 2011–2013 was used to estimate energy intakes (kJ) and total amount of food intakes (g), and to group items as beverages or non-beverages for different eating patterns [28]. For the current analysis, data from the first recall was used to preserve the large and nationally-representativeness sample across multiple age groups. However, to examine the robustness of findings, data from the second recall was also examined.

### Eating occasions and their definitions

During the dietary recall, participants and/or proxies recalled the starting clock time and selected the type of each EO (response options included: breakfast; brunch; lunch; dinner; supper; snack; morning tea; afternoon tea; drink/beverage; extended consumption; other/don’t know) that took place in the previous 24 h. Detail of the foods and beverages reported at each EO, including the amount that was consumed, was provided. Table 1 describes the eight definitions that were applied to determine EO. These were based on previously published definitions used in studies of children or adolescents [14, 17–20]. Two of the definitions classified EO into meals and snacks based on participant-identification of the EO type or the time-of-day [14, 17]. The remaining six definitions used a neutral approach whereby meals and snack labels were not assigned to specific EO. Instead, these neutral definitions identified individual EO using time and energy-based criteria (i.e., neutral definitions) [17–20].

**Table 1:** A summary of the eight definitions that were applied to determine eating occasions from dietary data collected in the NNPAS 2011–12

Definition applied	Description
*Meals and snacks*	
1) Participant-identified	Meal: Eating occasions reported by respondents as breakfast, brunch, lunch, dinner, and supper Snack: Eating occasions reported by respondents as snack, morning/afternoon tea and beverage break.
2) Time-of-day	Meal: Largest eating occasion (kJ) occurring between 05:30–9:30; 11:30–14:30 & 17:00–21:30 h Snack: All other eating occasions
*Neutral eating occasions*	
3) 15-min time interval	An individual eating occasion is separated in time from the preceding and succeeding EO by > 15 min.
4) 15-min time interval plus 21 kJ energy criterion	An individual eating occasion contains a minimum energy content of 21 kJ (5 kcal) and is separated in time from the preceding and succeeding eating occasions by > 15 min.
5) 15-min time interval plus 210 kJ energy criterion	An individual eating occasion contains a minimum energy content of 210 kJ (50 kcal) and is separated in time from the preceding and succeeding eating occasions by > 15 min.
6) 60-min time interval	An individual eating occasion is separated in time from the preceding and succeeding eating occasions by > 60 min.
7) 60-min time interval plus 21 kJ energy criterion	An individual eating occasion contains a minimum energy content of 21 kJ (5 kcal) and is separated in time from the preceding and succeeding eating occasions by > 60 min.
8) 60-min time interval plus 210 kJ energy criterion	An individual eating occasions contains a minimum energy content of 210 kJ (50 kcal) and is separated in time from the preceding and succeeding eating occasions by > 60 min.

Abbreviations: *NNPAS* National Nutrition and Physical Activity Survey

### Classification of meals and snacks

The participant-identified and time-of-day definitions were applied to classify meals and snacks, respectively (Table 1). Before applying these definitions, snacks and meals of the same type (i.e., as reported by participants) occurring ≤15 min of each other, respectively, were combined, consistent with previous studies [18, 21]. For example, two snacks reported at 10:00 am and 10:10 am would become one snack EO, commencing at 10:00 am, and two lunches reported at 12:15 pm and 12:30 pm would become one lunch EO commencing at 12:15 pm. EO that participants identified as breakfast, brunch, lunch, dinner, or supper were considered meals. Snacks included EO identified as snack, morning tea, afternoon tea or beverage break. Morning tea and afternoon tea are generally considered snack occasions in Australia and New Zealand (originating from the UK, these EO labels traditionally involved drinking tea with small amounts of food). For the participant-identified definition, EO identified by participants from the response options as “extended consumption” or “other/don’t know” were considered meals or snacks if they occurred at the same time or within 15 min of an EO identified by the participant as a meal or snack (e.g. interpreted as a continuation of the preceding EO) [21]. All remaining extended consumption occasions (*n* = 1009 EO) were then coded as snacks as this response option was included by the ABS to capture occasions where eating/drinking was spread over time (i.e. grazing) [25]. For the time-of-day definition, meals (breakfast/brunch, lunch, and dinner/supper) were defined as the largest EO (in terms of kJ) occurring between hours of the day where the largest peaks of energy consumption have previously been reported in Australian children and adolescents [14]. All other smaller occasions and EO outside of these hours were considered snacks (Table 1).

### Identification of individual eating occasions

Six ‘neutral’ definitions were applied to delineate individual EO (Table 1). These definitions did not rely on participant-identification of a meal/snack or the time of day. They varied according to the time separating preceding and succeeding EO (15 [18] or 60 min [19]) and the minimum allowable energy content of the EO (0 kJ/0 kcal [20], 21 kJ/5 kcal [17] or 210 kJ/50 kcal [18]). For example, the 15-min time interval definition would combine all reported EO occurring ≤15 min (including water only EO). Following this, the 15-min time interval plus a 21 kJ (5 kcal) energy content definition would then disregard any remaining EO that contained less than 21 kJ (5 kcal) of energy.

### Eating pattern and dietary intake variables

The mean total frequency of meals and snacks, the mean total energy intake (kJ) from meals and snacks, and the mean total energy intake per meal and snack (e.g., total energy intake from all meals and snacks divided by total frequency of meals and snacks, respectively) were calculated using the participant-identified and time-of-day definitions. For all six neutral definitions, the mean total eating frequency, the mean time between EO (min), and the mean total energy intake (kJ) per EO (total energy intake from all EO divided by total frequency of EO) were calculated. The mean total frequency of beverage-only occasions and the mean total energy intake (kJ) from all beverage-only occasions were also calculated for each neutral definition. Finally, participants’ daily total energy intake (kJ) and total weight (g) of food and beverage intake were calculated.

### Anthropometrics

Height (cm) was measured to 1 decimal point using a portable stadiometer and weight (kg) was taken using digital scales. BMI-for-age z-scores were calculated in Stata 15.0 (Stata Inc., College Station, TX, USA; zanthro package) by applying the World Health Organization growth reference standards [28].

### Sociodemographic characteristics

For the present analysis, age (y) was categorised into the following age-groups in alignment with dietary recommendations [29]: 2–3 y; 4–8 y; 9–11 y, 12–14 y and 15-18y, and each participant’s country of birth was categorised by the ABS as Australia, other ‘mainly English speaking’ countries or all other countries. Area-level disadvantage was determined using the ABS’s Socio-Economic for Areas (SEIFA) Index of Relative Socio-Economic Disadvantage quintiles which ranged from one (most disadvantaged) to five (most advantaged).

### Analytic sample

Children and adolescents aged 2 to 18 years with data for the first 24-h dietary recall, who were not pregnant or breastfeeding (*n* = 2 excluded) and who did not undertake shift work (e.g., night or rotating work shifts that may lead to altered eating patterns) in the past 4 weeks (*n* = 78 adolescents aged 15–18 years excluded) were eligible for inclusion in the present study (*n* = 2732 eligible; Additional File 1). Participants were excluded from the analysis (*n* = 31 excluded) if: time of eating was not reported, reported daily energy intake (< 210 kJ) was implausible or if they reported an EO as “other/don’t know” (i.e., EO that could not be categorised as a meal or snack). After all exclusions (*n* = 111), the final analytic sample included 1364 boys and 1337 girls of whom 1124 (82%) and 1082 (81%) had available data for height and weight, respectively (Additional File 1).

### Statistical analysis

Person-specific weights, adjusted for probability of selection and non-response, were used to provide estimates relating to the whole population and replicate weights (jack-knife delete 1 method) were used to account for the clustered survey design. All analyses were conducted in Stata 15 and were stratified by gender and age-group. Descriptive statistics for sociodemographic and eating patterns are presented as weighted proportions or weighted means (standard deviation). The F test (TEST command) with Bonferroni correction was used to determine differences between the different EO definitions. Alpha was conservatively set at 0.01 for comparisons between the participant-identified and time-of-day definition and at 0.003 for multiple comparisons between the six neutral definitions (0.05/15 comparisons). Mixed effect models were used to estimate intra-class correlation coefficients (ICC) for the absolute agreement of the frequency of meal and snack EO between the participant-identified and time-of-day definitions. Separate multiple linear regression models were used to determine the proportion of variance (model adjusted R-square value) in total energy intake (kJ), total amount of food/beverage intake (g) and BMI z-score (all continuous variables) predicted by the total meal and snack frequencies (both variables entered as separate predictors in the same model) and total EO frequency, calculated for each definition. The total energy intake and amount of food/beverage intake variables were positively skewed and therefore log-transformed prior to the regression analysis. The regression analysis with BMI z-score as the outcome included a crude model and a model additionally adjusted for total energy intake. To check the robustness of eating patterns determined using one dietary recall, all data management procedures and analyses were repeated using data from the second dietary recall that was completed by 822 boys and 792 girls, of whom 717 and 673 had available data on BMI, respectively. Due to the smaller number of participants who completed the second recall day, sensitivity analyses were stratified by gender and following age groups: < 12 years and 12 years and over.

## Results

Table 2 presents the sociodemographic characteristics of boy and girl participants from the NNPAS. There were no differences in proportions of boys and girls represented by age group, country of birth or area-level disadvantage. However, gender differences in total energy intake (kJ) and weight of food and beverage intake were observed for children aged 4–8 and 15–18 years. Total energy intake, but not weight of food and beverage intake, was also different between boys and girls aged 12–14 y (*p* < 0.01). There were no statistically significant gender differences in BMI-for-age z-scores.

**Table 2:** Sociodemographic characteristics of child and adolescent participants in the NNPAS 2011–12. Results are presented as weighted percentages (%) or weighted means (SD)

	Boys (***n*** = 1364)	Girls (***n*** = 1337)
**Age group (years), %**		
2–3	12	12
4–8	30	29
9–11	18	19
12–14	14	14
15–18	26	25
**Country of Birth, %**		
Australia	91	90
Other mainly English-speaking countries	5	4
All other countries	4	6
**Mainly speaks English at home, %**	93	94
**Area-level disadvantage (SEIFA)**^***1***^ **quintiles. %**		
Quintile 1	18	16
Quintile 2	20	18
Quintile 3	19	22
Quintile 4	17	20
Quintile 5	25	24
**Total energy intake (kJ), mean (SD)**		
*2–3 y*	6040 (2394)	5850 (2387)
*4–8 y*	7639 (2322)	6436 (2066)*
*9–11 y*	8962 (2830)	8100 (2602)
*12–14 y*	9590 (3136)	7832 (2813)*
*15–18 y*	10,270 (4210)	8178 (3173)*
**Total weight of food and beverage intake (g), mean (SD)**		
*2–3 y*	1873 (788)	1679 (713)
*4–8 y*	2162 (715)	1941 (605)*
*9–11 y*	2527 (801)	2384 (743)
*12–14 y*	2700 (868)	2500 (784)
*15–18 y*	3150 (1354)	2644 (946)*
**BMI-for-age z-scores, mean (SD)**		
*2–3 y*	0.8 (1.3)	0.8 (1.2)
*4–8 y*	0.7 (1.2)	0.6 (1.2)
*9–11 y*	1.1 (1.2)	0.7 (1.0)
*12–14 y*	0.6 (1.1)	0.8 (1.0)
*15–18 y*	0.5 (1.1)	0.3 (1.0)

Abbreviations: *BMI* body mass index; *NNPAS* National Nutrition and Physical Activity Survey

^1^Australian Bureau of Statistics Socio-Economic Indexes for Areas. SEIFA quintiles range from one (most disadvantaged) to five (most advantaged)

*P < 0.01; F test of significant difference between boys and girls with Bonferroni adjustment

### Characterisation of meals and snacks

When comparing the participant-identified and time-of-day definitions, differences were found for EO frequency of meals and snacks separately, but not for total EO, across all age-groups (Table 3). However, fewer differences were found for the total energy intake from either meals or snacks in boys (4–8 y, 9–11 y and 15–18 y only) and girls (12–14 y and 15–18 y only) between definitions. Regarding total energy intake per meal, differences were observed among boys aged 4–8 y and 9-11y and girls aged 2–3 y and 4–8 y. There were no differences in energy intake per snack when applying the participant-identified definition versus the time-of-day definition (Table 3).

**Table 3:** Total frequency of meals and snacks, total energy intake from meals and snacks, and total energy intake per meal and per snack for the participant-identified versus time-of-day definitions: results from 2011 to 12 NNPAS by gender and age groups^1^

	***n***	Boys		***n***	Girls	
		Participant-identified	Time-of-day		Participant-identified	Time-of-day
*Frequency of meals*						
*2–3 y*	226	3.0 (0.7)	2.8 (0.4)*	234	3.2 (0.8)	2.9 (0.4)*
*4–8 y*	393	3.1 (0.5)	2.9 (0.3)*	386	3.1 (0.5)	2.9 (0.3)*
*9–11 y*	223	3.1 (0.6)	2.9 (0.3)*	229	3.1 (0.5)	2.9 (0.3)*
*12–14 y*	159	3.0 (0.5)	2.8 (0.3)*	166	2.8 (0.7)	2.6 (0.5)*
*15–18 y*	363	2.7 (0.7)	2.6 (0.6)*	322	2.9 (0.6)	2.6 (0.6)*
*Frequency of snacks*						
*2–3 y*	226	3.8 (2.0)	4.0 (2.0)*	234	3.8 (2.2)	4.2 (2.3)*
*4–8 y*	393	3.4 (1.6)	3.6 (1.7)*	386	3.4 (1.4)	3.5 (1.5)*
*9–11 y*	223	3.4 (1.4)	3.6 (1.5)*	229	3.4 (1.3)	3.6 (1.2)*
*12–14 y*	159	3.1 (1.5)	3.3 (1.4)*	166	3.3 (1.5)	3.5 (1.4)*
*15–18 y*	363	3.0 (1.6)	3.1 (1.6)*	322	2.7 (1.5)	3.0 (1.6)*
*Total energy intake from meals (kJ)*						
*2–3 y*	226	4063 (2017)	3972 (1882)	234	3748 (1815)	3707 (1743)
*4–8 y*	393	5251 (1868)	5090 (1819)*	386	4331 (1663)	4314 (1650)
*9–11 y*	223	6069 (2151)	5836 (1968)*	229	5480 (2309)	5329 (2070)
*12–14 y*	159	6880 (2558)	6545 (2541)	166	5150 (2020)	4891 (1986)*
*15–18 y*	363	7252 (3380)	6822 (3181)*	322	5942 (2690)	5559 (2754)*
*Total energy intake from snacks (kJ)*						
*2–3 y*	226	1976 (1301)	2067 (1517)	234	2102 (1866)	2143 (1562)
*4–8 y*	393	2387 (1514)	2549 (1521)*	386	2104 (1339)	2122 (1241)
*9–11 y*	223	2893 (1859)	3126 (1856)*	229	2620 (1627)	2771 (1399)
*12–14 y*	159	2710 (2044)	3046 (2101)	166	2682 (1936)	2942 (1828)*
*15–18 y*	363	3018 (2577)	3448 (2933)*	322	2236 (1799)	2619 (1874)*
*Energy intake per meal (kJ)*^*2*^						
*2–3 y*	226	1372 (631)	1399 (630)	234	1188 (541)	1287 (567)*
*4–8 y*	393	1683 (574)	1741 (627)*	386	1413 (531)	1471 (548)*
*9–11 y*	223	1976 (748)	2081 (818)*	229	1790 (669)	1847 (706)
*12–14 y*	159	2377 (904)	2359 (890)	166	1849 (657)	1878 (684)
*15–18 y*	363	2673 (1148)	2689 (1267)	321	2115 (967)	2177 (1048)
*Energy intake per snack (kJ)*^*3*^						
*2–3 y*	225	547 (400)	542 (591)	231	548 (397)	538 (386)
*4–8 y*	387	753 (484)	758 (592)	381	675 (526)	647 (394)
*9–11 y*	220	899 (554)	908 (497)	225	802 (501)	832 (455)
*12–14 y*	153	919 (722)	953 (463)	162	865 (587)	867 (509)
*15–18 y*	351	1110 (1384)	1111 (976)	308	924 (776)	934 (659)

Abbreviations: *NNPAS* National Nutrition and Physical Activity Survey

^1^Values are all weighted means (SD). Total eating occasion frequency can be calculated for each definition by summing the frequency of meals and frequency of snacks

^*2*^n = 1 girl aged 15–18 y did not self-report eating any meals

^*3*^Self-reported snack consumers include *n* = 1336 boys and *n* = 1307 girls

^*^P < 0.01; F test of significant differences between definitions with Bonferroni adjustment

There was very good agreement (Intra-class Correlation coefficient [95% confidence interval]) in the frequency of snacks (boys: 0.93 [0.91, 0.94], girls: 0.93 [0.90, 0.94) between the participant-identified and time-of-day definitions. Results were similar after stratification by age group (boys: ICC range = 0.90–0.94; girls: ICC range = 0.89–0.94). However, poorer agreement between definitions was observed for the frequency of meals (boys: 0.36 [0.29, 0.43], girls: 0.38 [0.30, 0.46]). After stratification by age-group, ICCs ranged from 0.14–0.42 for boys and 0.13–0.51 for girls, with the lowest agreement observed for boys aged 4–8 y and girls aged 9–11 y (data not shown).

### Characterisation of eating occasions

Table 4 presents the total EO frequency, mean time between EOs, total energy intake per EO, total frequency of beverage-only occasions and total energy intake from beverage-only occasions for the six variations of the neutral definition. In general, frequency of EO (including beverage-only occasions) decreased and energy intake per EO increased concomitant with applying longer time intervals (15 to 60 min) to delineate individual EO. However, the decreases in EO frequency and increases in time between EO after applying a higher minimum energy criterion (21 to 210 kJ) were generally greater for the 15-min time interval than the 60-min time interval definition. Fewer differences between definitions were observed for frequency of beverage-only occasions and total energy intake from beverage-only occasions. Applying a 21 kJ or 210 kJ energy criterion to the 15-min time interval definition significantly reduced the frequency of beverage-only EO but had little impact on total energy intake from beverage-only occasions, when compared to no energy criterion. Few differences in frequency of beverage-only occasions and no differences in energy intake from beverage-only occasions were observed after applying each energy criterion within the 60-min interval definition. However, there were significant differences in total energy intake from beverage-only occasions when comparing variations of the 15-min time interval with variations of the 60-min time interval definitions.

**Table 4:** Total eating occasion frequency, mean time (minutes) between eating occasions across six neutral definitions, total energy intake (kJ) per eating occasion, total beverage-only eating occasion frequency and total energy intake (kJ) from beverage-only eating occasions: results from 2011 to 12 NNPAS by gender and age group^1^

			15-min	15-min + 21 kJ	15-min + 210 kJ	60-min	60-min + 21 kJ	60-min + 210 kJ
		n	Mean (SD)	Mean (SD)	Mean (SD)	Mean (SD)	Mean (SD)	Mean (SD)
*Eating occasion frequency*								
Boys	*2–3 y*	226	6.8 (2.1)	6.3 (2.0)	5.7 (1.8)	4.8 (1.1)	4.7 (1.1)	4.5 (1.2)
	*4–8 y*	393	6.5 (1.7)	6.1 (1.5)	5.6 (1.4)	4.9 (0.9)	4.8 (0.9)	4.6 (0.9)
	*9–11 y*	223	6.5 (1.5)	5.9 (1.3)	5.7 (1.2)	4.9 (0.9)	4.7 (0.9)	4.6 (0.9)
	*12–14 y*	159	6.0 (1.5)	5.4 (1.3)^a^	5.2 (1.2)^a^	4.8 (1.2)	4.5 (0.8)	4.4 (0.8)
	*15–18 y*	363	5.7 (1.7)	5.1 (1.5)	4.9 (1.5)	4.5 (1.3)	4.3 (1.2)	4.2 (1.2)
Girls	*2–3 y*	234	7.0 (2.4)	6.6 (2.3)	5.9 (2.1)	4.9 (0.9)	4.8 (1.3)	4.5 (1.3)
	*4–8 y*	386	6.5 (1.5)	6.0 (1.3)	5.6 (1.3)	5.0 (0.9)	4.8 (0.9)	4.6 (0.9)
	*9–11 y*	229	6.5 (1.3)	6.0 (1.1)	5.6 (1.1)	4.9 (0.9)	4.7 (0.9)	4.6 (0.9)
	*12–14 y*	166	6.1 (1.5)	5.5 (1.4)	5.3 (1.4)	4.8 (1.1)	4.5 (1.1)	4.4 (1.0)
	*15–18 y*	322	5.6 (1.7)	5.0 (1.4)	4.7 (1.3)^a^	4.5 (1.1)^a^	4.2 (1.0)	4.1 (1.0)
*Time between eating occasions (minutes)*								
Boys	*2–3 y*	226	131 (54)	142 (59)	158 (77)	187 (86)^a^	189 (87)^ab^	204 (101)^b^
	*4–8 y*	393	137 (39)	145 (63)	159 (49)	180 (48)^a^	183 (49)^a^	193 (55)
	*9–11 y*	223	140 (43)	152 (74)	158 (48)	188 (51)	193 (51)^a^	197 (52)^a^
	*12–14 y*	159	157 (45)	173 (66)^a^	178 (51)^a^	198 (56)	207 (57)^b^	212 (59)^b^
	*15–18 y*	363	166 (58)	184 (78)	190 (82)	213 (72)	223 (86)^a^	229 (91)^a^
Girls	*2–3 y*	234	132 (74)	141 (79)	160 (93)	186 (82)^a^	191 (85)^a^	204 (94)
	*4–8 y*	386	134 (44)	144 (45)	156 (48)	175 (48)	179 (48)	189 (52)
	*9–11 y*	229	137 (35)	148 (39)	159 (46)	184 (42)	190 (45)	198 (52)
	*12–14 y*	166	149 (45)	162 (49)^a^	168 (51)^a^	189 (44)	197 (44)^b^	201 (48)^b^
	*15–18 y*	322	175 (64)	192 (72)	201 (71)	220 (75)	228 (77)^a^	236 (83)^a^
*Energy intake (kJ) per eating occasion*								
Boys	*2–3 y*	226	921 (370)	996 (401)	1080 (374)	1278 (518)	1297(608)	1361 (521)
	*4–8 y*	393	1220 (408)	1301 (410)	1407 (442)	1592 (556)	1630 (745)	1704 (583)
	*9–11 y*	223	1431 (469)	1548 (493)	1599 (497)	1909 (712)	1953 (674)	1979 (694)
	*12–14 y*	159	1646 (555)	1824 (582)^a^	1879 (665)^a^	2072 (768)	2176 (618)^b^	2232 (834)^b^
	*15–18 y*	363	1888 (874)	2120 (957)	2172 (949)	2376 (1108)	2528 (1165)	2569 (1150)
Girls	*2–3 y*	234	868 (375)	927 (418)	1032 (406)	1235 (600)^a^	1264 (616)^a^	1337 (600)
	*4–8 y*	386	1037 (396)	1114 (397)	1191 (412)	1334 (477)	1369 (474)	1428 (497)
	*9–11 y*	229	1294 (475)	1402 (496)	1493 (502)	1701 (607)	1758 (609)	1828 (627)
	*12–14 y*	166	1314 (450)	1450 (478)	1505 (472)	1704 (665)	1811 (680)	1856 (687)
	*15–18 y*	322	1543 (646)	1716 (702)	1804 (698)^a^	1986 (1364)^a^	2097 (1362)	2165 (1375)
*Beverage-only occasion frequency*								
Boys	2–3 y	226	1.3 (1.2)	0.8 (1.0)	0.7 (1.0)	0.4 (0.8)	0.4 (0.7)^a^	0.3 (0.7)^a^
	4–8 y	393	0.8 (1.0)	0.4 (0.6)^a^	0.3 (0.6)^b^	0.3 (0.5)^ab^	0.1 (0.4)^c^	0.1 (0.3)^c^
	9–11 y	223	0.9 (1.0)	0.4 (0.6)^a^	0.3 (0.5)^a^	0.3 (0.6)^a^	0.2 (0.4)^b^	0.2 (0.4)^b^
	12–14 y	159	1.2 (1.0)	0.5 (0.7)^a^	0.5 (0.6)^ab^	0.6 (0.7)^a^	0.3 (0.5)^bc^	0.3 (0.5)^c^
	15–18 y	363	1.4 (1.1)	0.7 (0.8)^a^	0.7 (0.8)^a^	0.7 (0.8)^a^	0.4 (0.6)^b^	0.4 (0.6)^b^
Girls	2–3 y	234	1.3 (1.5)	0.9 (1.3)^a^	0.8 (1.2)^a^	0.5 (0.8)	0.4 (0.8)^b^	0.3 (0.8)^b^
	4–8 y	386	0.8 (0.9)	0.4 (0.6)^a^	0.3 (0.6)^b^	0.3 (0.5)^ab^	0.1 (0.4)^c^	0.1 (0.4)^c^
	9–11 y	229	0.9 (0.9)	0.4 (0.6)^a^	0.3 (0.6)^a^	0.4 (0.6)^a^	0.2 (0.4)^b^	0.2 (0.4)^b^
	12–14 y	166	1.1 (1.0)	0.5 (0.7)^a^	0.5 (0.6)^a^	0.6 (0.7)^a^	0.3 (0.5)^b^	0.3 (0.5)^b^
	15–18 y	322	1.1 (1.1)	0.5 (0.7)^a^	0.4 (0.7)^b^	0.6 (0.7)^ab^	0.3 (0.5)^c^	0.3 (0.5)^c^
*Energy intake (kJ) from beverage-only occasions*^*2*^								
Boys	2–3 y	226	515 (813)^a^	515 (813)^a^	508 (814)	246 (616)^b^	246 (616)^b^	244 (615)^b^
	4–8 y	393	232 (460)^a^	232 (460)^a^	226 (458)^a^	70 (200)^b^	70 (200)^b^	67 (200)^b^
	9–11 y	223	285 (470)^a^	285 (470)^a^	277 (469)^a^	145 (352)^b^	145 (352)^b^	143 (352)^b^
	12–14 y	159	348 (469)^a^	348 (469)^a^	340 (470)^ab^	223 (399)^b^	223 (399)^b^	220 (400)^b^
	15–18 y	363	635 (921)^a^	635 (920)^a^	633 (920)^a^	387 (745)^b^	387 (745)^b^	384 (746)^b^
Girls	2–3 y	234	528 (903)^a^	528 (903)^a^	509 (895)^a^	243 (626)^b^	243 (483)^b^	238 (625)^b^
	4–8 y	386	196 (402)^a^	196 (402)^a^	191 (404)	91 (288)^b^	91 (288)^b^	91 (288)^b^
	9–11 y	229	274 (519)^a^	274 (519)^a^	266 (520)^a^	185 (419)^b^	185 (419)^b^	182 (420)^b^
	12–14 y	166	389 (616)^a^	389 (616)^a^	383 (614)^a^	214 (463)^b^	214 (463)^b^	211 (463)^b^
	15–18 y	322	400 (850)^a^	400 (850)^a^	390 (851)	283 (773)^b^	283 (772)^b^	278 (772)^b^

Abbreviations: *NNPAS* National Nutrition and Physical Activity Survey. ^1^Pairwise comparisons between definitions using *F tests with Bonferroni correction; significance set at P* < 0.003. Estimates that share the same superscript letter are not significantly different (*P* > 0.003)

^2^Very small differences in mean energy intake from beverages-only occasions were observed between the 15-min and 15-min + 21 kJ (5 kcal) definitions which are not shown in the table due to the rounding up/down of decimal numbers to the nearest whole number

### Proportion of variance in total energy intake, total weight of food and beverage intake and BMI-for-age z-scores explained by the eating occasion definitions

Among both genders and across all age groups, the highest proportion of variance in total energy intake was predicted by the 15-min plus minimum 210 kJ neutral definition (range = 8.1–34.8; Table 5). No consistent finding was observed for total weight of food/beverage intake. The highest R-squared values were observed for the participant-identified definition among boys aged 15–18 y (16.2%) and for the time-of-day definition among girls aged 12–14 y (16.5%). The participant-identified definition best predicted variance in amount of food intake among boys and girls aged 15–18 years, whereas the time-of-day approach best predicted variance in amount of food intake among boys and girls aged 4–8 y, boys aged 9–11 y and girls aged 12–14 y. Table 6 presents the proportion of variance of BMI z-score predicted by the eight definition variations. Among boys across three age-groups, the participant-identified definition explained a higher proportion of variance of BMI z-score, whereas the same pattern was observed for girls when applying the time-of-day definition. Overall, model adjustment for total energy intake did not materially alter these results, except for boys aged 2–3 y.

**Table 5:** Proportion of variance (%) of total energy intake (kJ) and total weight (g) of food and beverage intake predicted by total meal or snack frequency and total eating occasion frequency: comparison of eight eating occasion definitions: results from 2011 to 12 NNPAS by gender and age group^1^

		Participant-identified		Time-of-day		15-min		15-min + 21 kJ		15-min + 210 kJ		60-min		60-min + 21 kJ		60-min + 210 kJ	
		Total meal and snack frequency^2^				Total eating occasion frequency											
	*n*	*EI*	*Wgt*	*EI*	*Wgt*	*EI*	*Wgt*	*EI*	*Wgt*	*EI*	*Wgt*	*EI*	*Wgt*	*EI*	*Wgt*	*EI*	*Wgt*
**Boys**																	
*2–3 y*	226	22.8	14.0	20.1	12.9	19.7	11.6	19.4	12.4	**33.5**	13.5	13.7	11.1	15.7	11.5	21.6	**14.7**
*4–8 y*	393	14.1	13.2	13.7	**13.3**	13.6	13.2	15.2	12.3	**16.8**	12.4	4.3	3.8	5.1	4.4	7.7	4.6
*9–11 y*	223	15.6	7.6	14.1	**11.0**	13.6	7.3	17.1	6.6	**22.0**	7.5	4.1	4.3	6.1	4.2	10.2	5.5
*12–14 y*	159	14.2	8.7	18.2	8.7	14.2	8.4	18.6	8.7	**19.6**	**10.1**	4.9	0.8	13.9	2.2	11.0	2.4
*15–18 y*	363	21.3	**16.2**	13.1	13.5	13.0	13.0	16.9	7.2	**21.2**	7.5	4.4	4.2	11.8	5.3	16.1	5.6
**Girls**																	
*2–3 y*	234	19.5	11.7	17.7	11.7	17.2	11.6	16.9	9.3	**30.7**	**13.1**	6.1	6.3	6.2	4.7	17.5	8.8
*4–8 y*	386	3.4	2.6	3.5	**6.1**	3.2	2.6	5.6	2.3	**10.5**	4.2	2.0	2.6	3.6	3.2	4.9	4.2
*9–11 y*	229	1.9	1.1	1.9	0.5	1.7	0.4	2.7	0.3	**8.1**	0.1	1.5	**3.5**	2.8	2.4	5.9	1.4
*12–14 y*	166	23.5	15.5	21.8	**16.5**	21.6	12.7	29.0	7.2	**34.8**	10.0	3.6	5.3	9.8	4.7	11.2	6.4
*15–18 y*	322	8.5	**15.8**	7.9	15.4	7.8	15.4	9.1	10.1	**14.0**	11.2	1.4	6.7	4.5	8.9	6.6	9.3

Abbreviations: *EI* energy intake; *NNPAS* National Nutrition and Physical Activity Survey; *wgt* weight

^1^Values are R^2^ from linear regression models stratified by gender and age group. Highest values are highlighted in bold

^2^Meal and snack frequency entered as separate variables in the linear regression models

**Table 6:** Proportion of variance (%) of BMI z-score predicted by each eating occasion definition: results from 2011 to 12 NNPAS by gender and age group^*1*^

		Participant-identified		Time-of-day		15 min		15-min + 21 kJ		15-min + 210 kJ		60-min		60-min + 21 kJ		60-min + 210 kJ	
		Total meal and snack frequency^2^				Total eating occasion frequency											
	*n*	*crude*	*adj*	*crude*	*adj*	*crude*	*adj*	*crude*	*adj*	*crude*	*adj*	*crude*	*adj*	*crude*	*adj*	*crude*	*adj*
**Boys**																	
*2–3 y*	171	**3.0**	4.5	1.6	3.9	1.6	3.7	1.3	3.6	0.2	4.0	0.3	5.2	0.2	5.2	0.7	**7.3**
*4–8 y*	319	< 0.1	0.2	< 0.1	0.2	< 0.1	0.2	0	0.2	< 0.1	0.3	**2.3**	**2.3**	1.8	1.8	1.1	1.1
*9–11 y*	189	**3.1**	**3.2**	2.7	2.7	2.2	2.2	2.4	2.4	1.3	1.3	0.2	0.4	0	0.3	< 0.1	0.3
*12–14 y*	133	**5.3**	**5.5**	3.4	3.5	2.8	3.0	2.2	2.4	2.3	2.6	0.6	1.5	1.4	2.0	1.8	2.3
*15–18 y*	312	0.3	0.4	**0.6**	**0.7**	0.1	0.2	< 0.1	< 0.1	0	< 0.1	< 0.1	0.1	< 0.1	0.1	< 0.1	0.1
**Girls**																	
*2–3 y*	182	1.5	3.5	**2.7**	**4.6**	1.1	3.4	1.6	4.1	0.2	2.3	0.3	1.1	< 0.1	1.0	0.3	1.0
*4–8 y*	300	0.3	0.4	**2.3**	**2.4**	0.2	0.3	0.2	0.3	0.3	0.4	1.1	1.1	1.6	1.6	1.3	1.3
*9–11 y*	201	1.9	3.0	**3.5**	**4.5**	1.6	2.7	1.3	2.4	1.5	2.3	0.4	2.1	> 0.1	1.4	0.2	1.5
*12–14 y*	141	**2.9**	6.6	2.6	**6.9**	2.2	6.3	1.2	5.9	0.3	4.0	4.4	6.7	2.3	5.3	2.4	5.6
*15–18 y*	258	**2.3**	**3.0**	1.1	2.0	0.2	1.0	0.4	1.1	0.2	1.0	2.0	2.7	1.4	2.0	1.3	1.9

Abbreviations: *Adj* adjusted; *BMI* body mass index; *NNPAS* National Nutrition and Physical Activity Survey

^1^Values are R^2^ from linear regression models stratified by gender and age group, unadjusted (i.e., crude) and adjusted for total energy intake. Highest values for crude and adjusted models are highlighted in bold

^*2*^Meal and snack frequency entered as separate variables in the linear regression models

### Sensitivity analysis: characterisation of eating patterns using the second dietary recall day

Additional File 2 presents the sociodemographic characteristics of boys and girls who completed the second dietary recall. Overall, reported total energy intakes were significantly lower on the second recall day when compared to the first day, except for girls aged < 12 years (t-test with unequal variances, *p* < 0.01). There were no significant differences in BMI z-scores between days one and two (*p* > 0.05). Whilst there were some small quantitative differences in the estimates of the eating pattern variables between dietary recall days, the main study findings were similar (Additional Files 3 to 6).

## Discussion

To our knowledge, this is the first study to objectively compare a wide range of previously published EO definitions in relation to the characterization of eating patterns in children and adolescents aged 2 to 18 years old. The degree to which these definitions predicted the proportion of variance in total energy intake, weight of food and beverage intake and BMI-for-age z-scores were also examined. Small differences were consistently found in the frequency of meals and snacks and total EO when comparing the participant-identified with the time-of-day definition and across the six variations of the neutral definitions, respectively. Other characteristics (e.g., total energy intake from meals/snacks, time between EO and energy intake per EO) also differed but were not consistent across all definitions and age groups. The neutral definition with a 15-min time interval plus 210 kJ criterion best predicted variance in total energy intake among both genders and across all age groups. However, no consistent finding was observed for weight of food and beverage intake, and the time-of-day and participant-identified definitions generally performed better than the neutral definitions when predicting variance in BMI z-scores. Given a variety of definitions have been applied in studies of children’s eating patterns to date, this methodologic study provides evidence that the choice of EO definition should consider the population age and research outcome under study. For example, our findings suggest EO definitions with large time intervals may underestimate eating frequency in young children and definitions that delineate meals and snacks may be more appropriate when BMI is the outcome of interest.

Studies comparing EO definitions in young populations are rare [4, 30]. In line with our findings, Llaurado et al. applied a 15-min time interval to define EO and observed a reduction in the mean number of total EO per day (7.5 vs 6.2) and stronger associations with energy intake for each additional EO (88 vs 218 kJ) after excluding EO less than 210 kJ in UK adolescents aged 11–18 y [4]. Murakami and Livingstone found small differences in the frequency of meals and snacks calculated using a participant-identified versus a time-of-day approach, in a nationally representative sample of US children and adolescents [30]. Associations for meal and snack frequency with total energy intake were also stronger for the participant-identified definition, which applied the same 15-min time interval as the present study. In a similar methodological study of Australian adults, the 15-min time interval plus a 210 kJ energy criterion best predicted variance in total energy intake and the participant-identified also performed better than the time-of-day definition [21]. These findings suggest that the 15-min interval plus an energy criterion of 210 kJ may best capture the energy contribution of EO to overall dietary intake. This finding may be explained by the variability, or noise, in the EO frequency data introduced by the inclusion of EOs containing non-energy or low-energy foods and beverages when lower or no energy criteria are applied [31].

Beverages may also be a source of variability in the estimation of the total weight of food and beverage consumption and thus explain our inconsistent finding for this outcome [31]. Energy density, or kJ per gram of food and beverage consumption, may be a more relevant outcome to examine due to its associations with dietary intake and BMI [32–35]. Further, studies in adults [36] and adolescents [34] suggest that the energy density of foods but not beverages are predictive of weight status and future research should examine the relationship between EO frequency and energy density, using approaches that include and exclude beverages.

In the present study, applying a 21 kJ energy criterion significantly reduced the frequency of beverage-only EO but not the energy intake from beverage-only occasions among both genders and across all age groups. Applying a 210 kJ criterion had little further impact on beverage-only frequency or energy intake from beverage-only EO whereas application of a 60-min time interval substantially reduced both estimates. This suggests the 21 kJ is sufficient to remove non and very low energy beverages, whereas a 60-min time interval is unlikely to capture EO where higher energy beverages are consumed on their own. Therefore, a 15-min plus a 21 kJ energy criterion neutral may be most appropriate if the research goal is to examine frequency of, and energy intake from, beverage-only EO in the context of children’s eating patterns. However, no energy criterion is needed if the goal is to capture non and low-energy beverage-only EO (e.g., water or herbal tea).

Currently, interpreting the discrepant literature on EO frequency and obesity in young people is complicated by a lack of consensus about EO definitions. In the present study, definitions that delineated meals and snacks were stronger predictors of BMI z-scores than the neutral definitions. This may be because food groups eaten as snacks tend to differ from those eaten at meals [12, 37]. In support of this finding, the association with BMI percentile among US children aged 6-11y was stronger for snack frequency, based on participant-identified (β = 2.82) and time-of-day (β = 2·52) definitions, than overall EO frequency using a neutral approach (β = 2.09). In the same study, the delineation of meals and snacks also influenced associations with BMI among adolescents aged 12–19 y and these associations varied according to the definition employed [30]. Further, studies in adult populations support the differentiation of EO frequency by meals and snacks in obesity research [38–40]. Given these findings and the hypothesised different effects of meals and snacks on dietary intakes and adiposity [4, 30, 41], a definition that categorises meals and snacks using a participant identified or time-of-day approach may be suitable choices for use in children’s eating patterns and obesity research.

In line with previous research [14, 18, 19, 30], this study found total EO frequency and snack frequency decreased with increasing age, irrespective of the definition employed. When comparing definitions with 15-min and 60-min time intervals, we found the magnitude of difference in total EO frequency and relative contribution to energy intake decreased with increasing age intervals for both boys and girls. For example, a 60-min time interval resulted in a 2–2.1 lower EO frequency and a 39–42% increase in energy intake per EO for children aged 2–3 years compared to 1.2–1.4 and 26–29% in those aged 15–18 years, respectively. Applying a 210 kJ energy criteria to the 15-min interval showed a similar age trend but the magnitude of difference was smaller. Nonetheless, these findings suggest that EO definitions with a shorter time interval and a lower energy criterion may be more appropriate for characterising young children’s eating patterns. The findings also suggest that analysing children’s eating pattern by age group may be more important than by gender and should be considered in future research.

The present study found only small quantitative differences in meal and snack frequencies and their relative contributions to total energy intake when comparing the participant-identified and time-of-day definitions. The agreement between these two definitions was good for snack but not meal frequencies. This may be explained by the rigidness of the time-of-day definition which only captures meals eaten at the conventional time windows of breakfast, lunch, and dinner; meals eaten outside of these times are not captured. Whilst differences in the quantitative estimates of energy intake from meals and snacks were small in the present study, when comparing the participant-identified and time-of-day definitions, further methodologic research examining these definitions on the characterisation of children’s dietary intakes is needed to understand the different effects of meals and snacks on food and nutrient intakes. For instance, snacking is recommended for very active and/or growing children to meet energy and nutrient requirements [42]; understanding the dietary contribution of snacks (as distinct from meals) to diet quality and health will be important for the development of dietary guidance of food intakes across different EO and developmental stages [11]. Finally, the present analysis focussed on definitions that have been used to delineate individual EO in studies examining children’s meals, snack or EO frequency. Other EO parameters such EO size (kJ per EO) and EO energy density (kJ per g per EO) have been previously linked to children’s dietary intake and weight status and could be considered in future research that investigates how to best assess children’s eating patterns [33–35].

Some strengths of the present study include the objective comparison of eight EO definitions previously applied in the eating patterns literature among a nationally representative sample of Australian children and adolescents. Eating patterns calculated for each definition were also examined in relation to objective measures of BMI and the large sample size allowed exploration of gender and age group differences. A limitation of our study is the use of a single 24-h dietary recall to estimate children’s eating patterns which is unlikely to capture day-to-day variation. However, the study findings did not materially differ after analysing data from the subset of participant who completed the second dietary recall. Nonetheless, more recall days may be needed to better understand the variability in children’s eating patterns. In the present study, detailed information about participants’ ethnicity was not available and the results may not be generalisable to children and adolescent populations with different ethnic backgrounds or from other countries. Previous research has shown that eating patterns are influenced by cultural factors and research in populations with different ethnic backgrounds is warranted [43, 44].

## Conclusions

In summary, variations in the definition of EO affect how eating patterns are characterized, particularly in young children. Whilst a variation of the neutral definition (15-min time interval plus 210 kJ energy criterion) best predicted variance in total energy intake, definitions that delineated meals and snack performed better in relation to BMI. This study also found that applying a 21 kJ energy criterion to the neutral definition (15-min time interval) was sufficient to remove non and very low energy beverages whereas a 60-min time interval was unlikely to capture EO in which higher energy beverages were consumed. These findings suggest the choice of EO definition may depend on the research outcomes of interest (e.g., beverage-only EO, total energy intake or BMI), rather than a one-size fits all approach. Smaller time intervals of 15 min and energy criteria of 21 kJ may also be more appropriate for capturing dietary intakes at distinct EO among younger children, particularly those aged 2–3 y. However, further studies in children from other countries that examine how different EO definitions affect associations with food intakes, diet quality and other health outcomes are needed to support these findings and to inform a standard approach.

## Supplementary Information

**Additional file 1.**

**Additional file 2.**

**Additional file 3.**

**Additional file 4.**

**Additional file 5.**

## Data Availability

Data are available on request from the Australian Bureau of Statistics from https://www.abs.gov.au/websitedbs/D3310114.nsf/home/Microdata%2BEntry%2BPage. The research team does not have the permission to release the data to third parties.

## Abbreviations

ABS: Australian Bureau of Statistics
AUSNUT: Australian Food and Nutrient Database
BMI: Body mass index
EI: Energy intake
EO: Eating occasion
ICC: Intra-class correlation coefficient
NNPAS: National Nutrition and Physical Activity Survey
SD: Standard deviation
wgt: Weight
