# Correction: Characterizing children’s eating patterns: does the choice of eating occasion definition matter?

**DOI:** 10.1186/s12966-022-01402-0

**Published:** 2023-01-11

**Authors:** Rebecca M. Leech, Alison C. Spence, Kathleen E. Lacy, Miaobing Zheng, Anna Timperio, Sarah A. McNaughton

**Affiliations:** Institute for Physical Activity and Nutrition, Deakin University, Geelong, Victoria Australia

**Correction: Int J Behav Nutr Phys Act 18, 165 (2021)**

**https://doi.org/10.1186/s12966-021-01231-7**

Following publication of the original article [1], the authors would like to correct the age group labels in the text, tables, and additional file 2.

The incorrect age group labels are: 12–14 years and 15–18 years.

The correct age group labels are: 12–13 years and 14–18 years.

The original article [1] has been corrected.
