# Supplementary material for: Characterizing children’s eating patterns: does the choice of eating occasion definition matter?
Source: Int J Behav Nutr Phys Act. 2021 Dec 19;18:165. doi: 10.1186/s12966-021-01231-7 (PMC8684678; doi:10.1186/s12966-021-01231-7)
Supplement: Supplementary file 1 — Additional file 1. [file 12966_2021_1231_MOESM1_ESM.docx]

**Additional File 1.** Participant flowchart for inclusion in the analysis of children’s eating patterns
