# Supplementary material for: Characterizing children’s eating patterns: does the choice of eating occasion definition matter?
Source: Int J Behav Nutr Phys Act. 2021 Dec 19;18:165. doi: 10.1186/s12966-021-01231-7 (PMC8684678; doi:10.1186/s12966-021-01231-7)
Supplement: Supplementary file 2 — Additional file 2. [file 12966_2021_1231_MOESM2_ESM.docx]

**Additional File 2** Sociodemographic characteristics of children and adolescents who completed the second dietary recall in the NNPAS 2011-12. Results are presented as weighted percentages (%) or weighted means (SD).

|  | **Boys (n=822)** | **Girls (n=792)** |
| --- | --- | --- |
| **Age group (years), %** |  |  |
| 2-3 | 11 | 13 |
| 4-8 | 31 | 27 |
| 9-11 | 18 | 19 |
| 12-14 | 13 | 14 |
| 15-18 | 25 | 27 |
| **Country of Birth, %** |  |  |
| Australia | 92 | 91 |
| Other mainly English-speaking countries | 4 | 4 |
| All other countries | 4 | 5 |
| **Mainly speaks English at home, %** | 93 | 94 |
| **Area-level disadvantage (SEIFA)*^1^* quintiles. %** |  |  |
| Quintile 1 | 17 | 14 |
| Quintile 2 | 18 | 20 |
| Quintile 3 | 20 | 20 |
| Quintile 4 | 16 | 19 |
| Quintile 5 | 28 | 26 |
| **Total energy intake (kJ), mean (SD)** |  |  |
| *2-3 y* | 6037 (2457) | 5502 (2387) |
| *4-8 y* | 7183 (2181) | 6196 (2066)* |
| *9-11 y* | 8049 (2747) | 7673 (2602) |
| *12-14 y* | 8838 (2764) | 7841 (2813) |
| *15-18 y* | 10162 (4327) | 7147 (3173)* |
| **Total weight of food and beverage intake (g), mean (SD)** |  |  |
| *2-3 y* | 1718 (744) | 1743 (841) |
| *4-8 y* | 2032 (584) | 2043 (893) |
| *9-11 y* | 2513 (1041) | 2341 (618) |
| *12-14 y* | 2688 (1033) | 2304 (727) |
| *15-18 y* | 3102 (1412) | 2494 (819)* |
| **BMI-for-age z-scores, mean (SD)** |  |  |
| *2-3 y* | 0.8 (1.3) | 1.0 (1.3) |
| *4-8 y* | 0.5 (1.1) | 0.6 (1.2) |
| *9-11 y* | 0.9 (1.2) | 0.6 (1.0) |
| *12-14 y* | 0.5 (1.0) | 0.8 (1.0) |
| *15-18 y* | 0.5 (1.2) | 0.2 (1.0) |

Abbreviations: BMI, body mass index; NNPAS, National Nutrition and Physical Activity Survey

*^1^*Australian Bureau of Statistics Socio-Economic Indexes for Areas. SEIFA quintiles range from one (least disadvantaged) to five (most disadvantaged).

*P<0.01; F test of significant difference between boys and girls with Bonferroni adjustment
