# Supplementary material for: Characterizing children’s eating patterns: does the choice of eating occasion definition matter?
Source: Int J Behav Nutr Phys Act. 2021 Dec 19;18:165. doi: 10.1186/s12966-021-01231-7 (PMC8684678; doi:10.1186/s12966-021-01231-7)
Supplement: Supplementary file 3 — Additional file 3. [file 12966_2021_1231_MOESM3_ESM.docx]

| **Additional File 3** Total frequency of meals and snacks and total energy intake from meals and snacks for the participant-identified versus time-of-day definitions: results from 2011-12 NNPAS participants who completed the second dietary recall.^1^ | | | | | | | | |  |
| --- | --- | --- | --- | --- | --- | --- | --- | --- | --- |
|  | ***n*** | **Boys** | | | ***n*** | | **Girls** | | |
|  |  | Participant-identified | Time-of-day |  | | Participant-identified | | Time-of-day |  |
| *Frequency of meals* | | | | | |  | |  |  |
| *<12 y* | 502 | 3.0 (0.5) | 2.9 (0.4)* | 499 | | 3.0 (0.6) | | 2.9 (0.4)* |  |
| *≥12 y* | 320 | 2.9 (0.5) | 2.8 (0.5)* | 293 | | 2.8 (0.7) | | 2.7 (0.6) |  |
| *Frequency of snacks* | | | | | |  | |  |  |
| <12 y | 502 | 2.9 (1.4) | 3.0 (1.4)* | 499 | | 3.0 (1.4) | | 3.2 (1.5)* |  |
| ≥12 y | 320 | 2.5 (1.4) | 2.7 (1.4)* | 293 | | 2.3 (1.4) | | 2.4 (1.4) |  |
| *Total energy intake from meals (kJ)* | | |  |  | |  | |  |  |
| <12 y | 502 | 5221 (2216) | 5078 (2144) | 499 | | 4454 (1934) | | 4353 (1830) |  |
| ≥12 y | 320 | 7095 (2918) | 6712 (2785)* | 293 | | 5443 (2362) | | 5310 (2423) |  |
| *Total energy intake from snacks (kJ)* | | |  |  | |  | |  |  |
| <12 y | 502 | 2006 (1474) | 2152 (1545) | 499 | | 2072 (1375) | | 2178 (1378) |  |
| ≥12 y | 320 | 2615 (2660) | 2998 (2432)* | 293 | | 1918 (1955) | | 2074 (1866) |  |

Abbreviations: NNPAS, National Nutrition and Physical Activity Survey

^1^Values are all weighted means (SD)

*^*^P<0.01; F* test of significant differences between definitions with Bonferroni adjustment
