# Supplementary material for: Characterizing children’s eating patterns: does the choice of eating occasion definition matter?
Source: Int J Behav Nutr Phys Act. 2021 Dec 19;18:165. doi: 10.1186/s12966-021-01231-7 (PMC8684678; doi:10.1186/s12966-021-01231-7)
Supplement: Supplementary file 4 — Additional file 4. [file 12966_2021_1231_MOESM4_ESM.docx]

| **Additional File 4.** Total eating occasion frequency, mean time (minutes) between eating occasions across six neutral definitions, and total energy intake (kJ) per eating occasion (kJ): results from 2011-12 NNPAS participants who completed the second dietary recall.^1^ | | | | | | | | |
| --- | --- | --- | --- | --- | --- | --- | --- | --- |
|  |  |  | **15-min** | **15-min + 21kJ** | **15 min + 210kJ** | **60-min** | **60-min + 21kJ** | **60-min + 210kJ** |
|  |  | n | Mean (SD) | Mean (SD) | Mean (SD) | Mean (SD) | Mean (SD) | Mean (SD) |
| *Eating occasion frequency* | | |  |  |  |  |  |  |
| Boys | <12 y | 502 | 5.9 (1.5) | 5.5 (1.4) | 5.2 (1.3) | 4.8 (1.0) | 4.7 (1.0) | 4.5 (1.0) |
|  | ≥12 y | 320 | 5.5 (1.5) | 4.9 (1.3) | 4.8 (1.3) | 4.5 (1.1) | 4.3 (1.1)^a^ | 4.3 (1.1)^a^ |
| Girls | <12 y | 499 | 6.1 (1.6) | 5.6 (1.5) | 5.3 (1.3) | 4.9 (1.0) | 4.7 (1.0) | 4.6 (1.0) |
|  | ≥12 y | 293 | 5.1 (1.6) | 4.6 (1.4)^a^ | 4.4 (1.4)^b^ | 4.4 (1.2)^ab^ | 4.1 (1.2) | 4.0 (1.1) |
| *Time between eating occasions (minutes)* | | | |  |  |  |  |  |
| Boys | <12 y | 502 | 149 (52) | 161 (58) | 170 (60) | 186 (59) | 192 (68) | 198 (70) |
|  | ≥12 y | 320 | 170 (57) | 191 (64)^a^ | 194 (65)^a^ | 211 (67) | 218 (71)^b^ | 221 (72)^b^ |
| Girls | <12 y | 499 | 149 (43) | 162 (61) | 173 (63) | 185 (69) | 192 (70) | 200 (73) |
|  | ≥12 y | 293 | 186 (80) | 207 (95)^a^ | 213 (94)^b^ | 220 (99)^ab^ | 232 (110) | 236 (109) |
| *Energy intake (kJ) per eating occasion* | | | |  |  |  |  |  |
| Boys | <12 y | 502 | 1279 (527) | 1370 (582) | 1435 (582) | 1556 (620) | 1592(634) | 1635 (635) |
|  | ≥12 y | 320 | 1844 (699) | 2093 (863) | 2121 (860) | 2268 (1008) | 2391 (1056) | 2417 (1052) |
| Girls | <12 y | 499 | 1124 (434) | 1221 (479) | 1286 (496) | 1379 (559) | 1433 (573) | 1476 (567) |
|  | ≥12 y | 293 | 1485 (600) | 1658 (681) | 1708 (673) | 1743 (877) | 1847 (882) | 1886 (878) |

Abbreviations: NNPAS, National Nutrition and Physical Activity Survey

^1^Pairwise comparisons between definitions using *F tests with Bonferroni correction; significance set at P*<0.003. Estimates that share the same superscript letter are not significantly different (P>0.003).
