# Supplementary material for: Characterizing children’s eating patterns: does the choice of eating occasion definition matter?
Source: Int J Behav Nutr Phys Act. 2021 Dec 19;18:165. doi: 10.1186/s12966-021-01231-7 (PMC8684678; doi:10.1186/s12966-021-01231-7)
Supplement: Supplementary file 5 — Additional file 5. [file 12966_2021_1231_MOESM5_ESM.docx]

| **Additional File 5.** Proportion of variance (%) of total energy intake (kJ) and total weight (g) of food and beverage intake predicted by total meal or snack frequency and total eating occasion frequency: results from 2011-12 NNPAS participants who completed the second dietary recall *^1^* | | | | | | | | | | | | | | | | | |
| --- | --- | --- | --- | --- | --- | --- | --- | --- | --- | --- | --- | --- | --- | --- | --- | --- | --- |
|  |  | **Participant-identified** | | **Time-of-day** | | **15-min** | | **15-min +**  **21 kJ** | | **15-min +**  **210 kJ** | | **60-min** | | **60-min +**  **21 kJ** | | **60-min +**  **210 kJ** | |
|  |  | Total meal and snack frequency^2^ | | | | Total eating occasion frequency | | | | | | | | | | | |
|  | *n* | *EI* | *Wgt* | *EI* | *Wgt* | *EI* | *Wgt* | *EI* | *Wgt* | *EI* | *Wgt* | *EI* | *Wgt* | *EI* | *Wgt* | *EI* | *Wgt* |
| **Boys** |  |  |  |  |  |  |  |  |  |  |  |  |  |  |  |  |  |
| <12 y | 502 | 4.9 | 11.1 | 5.2 | 13.4 | 3.8 | 10.1 | 5.2 | 8.3 | **9.8** | 13.8 | 3.8 | 12.2 | 5.8 | 12.5 | 9.2 | **17.7** |
| ≥12 y | 320 | 14.9 | **11.2** | 14.1 | 10.3 | 14.0 | 10.3 | 14.5 | 5.2 | **15.5** | 6.3 | 4.5 | 2.5 | 7.1 | 1.3 | 7.7 | 2.1 |
| **Girls** |  |  |  |  |  |  |  |  |  |  |  |  |  |  |  |  |  |
| <12 y | 499 | 8.6 | 10.7 | 9.1 | **11.3** | 8.6 | 10.1 | 7.1 | 7.1 | **11.0** | 9.0 | 6.0 | 8.9 | 5.1 | 7.8 | 7.6 | 8.7 |
| ≥12 y | 293 | 22.3 | 14.5 | 22.7 | **20.0** | 22.5 | 13.8 | 25.9 | 15.0 | **30.5** | 17.1 | 15.3 | 12.0 | 17.7 | 14.9 | 20.8 | 15.8 |

Abbreviations: EI, energy intake; NNPAS, National Nutrition and Physical Activity Survey; wgt, weight

*^1^*Values are R^2^ from linear regression models stratified by gender and age group. Highest values are highlighted in bold.

^2^Meal and snack frequency entered as separate variables in the linear regression models.

| **Additional File 6.** Proportion of variance (%) of BMI z-score predicted by each eating occasion definition: results from 2011-12 NNPAS participants who completed the second dietary recall.*^2^* | | | | | | | | | | | | | | | | | |
| --- | --- | --- | --- | --- | --- | --- | --- | --- | --- | --- | --- | --- | --- | --- | --- | --- | --- |
|  |  | **Participant-identified** | | **Time-of-day** | | **15-min** | | **15-min +**  **21 kJ** | | **15-min +**  **210 kJ** | | **60-min** | | **60-min +**  **21 kJ** | | **60-min +**  **210 kJ** | |
|  |  | Total meal and snack frequency^2^ | | | | Total eating occasion frequency | | | | | | | | | | | |
|  | *n* | *crude* | *adj* | *crude* | *adj* | *crude* | *adj* | *crude* | *adj* | *crude* | *adj* | *crude* | *adj* | *crude* | *adj* | *crude* | *adj* |
| **Boys** |  |  |  |  |  |  |  |  |  |  |  |  |  |  |  |  |  |
| <12 y | 425 | 0.3 | 0.8 | **1.1** | **1.3** | 0.2 | 0.6 | <0.1 | 0.4 | 0.1 | 0.6 | 0.2 | 0.6 | 0.1 | 0.5 | <0.1 | 0.5 |
| ≥12 y | 290 | 1.3 | 1.3 | **1.9** | **1.9** | <0.1 | 0.2 | 0.2 | 0.3 | 0.1 | 0.2 | 1.4 | 1.5 | **1.9** | 1.9 | 1.4 | 1.4 |
| **Girls** |  |  |  |  |  |  |  |  |  |  |  |  |  |  |  |  |  |
| <12 y | 418 | **1.8** | **2.8** | 1.6 | 2.6 | 1.5 | 2.5 | 1.0 | 2.2 | 1.0 | 2.1 | 1.1 | 2.3 | 0.7 | 2.1 | 1.0 | 2.2 |
| ≥12 y | 254 | **1.5** | **1.5** | 0.3 | 0.4 | 0.2 | 0.3 | <0.1 | 0.2 | <0.1 | 0.2 | 0.0 | 0.2 | 0.1 | 0.2 | 0.0 | 0.2 |

Abbreviations: NNPAS, National Nutrition and Physical Activity Survey

*^1^*Values are R^2^ from linear regression models stratified by gender and age group, unadjusted (i.e., crude) and adjusted for total energy intake. Highest values for crude and adjusted models are highlighted in bold.

*^2^*Meal and snack frequency entered as separate variables in the linear regression models.
